# Supplementary material for: The mlpt/Ubr3/Svb module comprises an ancient developmental switch for embryonic patterning
Source: eLife. 2019 Mar 21;8:e39748. doi: 10.7554/eLife.39748 (PMC6428570; doi:10.7554/eLife.39748)
Supplement: Supplementary file 1. — (A) Genes identified in the genome wide iBeetle RNAi screen that had phenotypes resembling those of mlpt. iB 00966 and 09278, had the most highly penetrant RNAi phenotype with the strongest resemblance to those of mlpt. NCBI annotates these as belonging to a single locus LOC657900 encoding a 6592 bp mRNA (accession number XM_964327) that corresponds to Tc-ubr3. (B) Oligonucleotides used in the manuscript. Nucleotides shown in red indicate tags of parts of T7 (3’ primer) and SP6 (5’ primer) promoter sequences attached to gene-specific sequences for nested PCR. Nucleotides shown in blue highlight minimal T7 promoter used in subsequent in vitro T7 RNA polymerase transcription. (C) Oligos used for generating Tc-svb CRISPR mutant. (D) Plasmids used for generating Tc-svb CRISPR mutant. [file elife-39748-supp1.doc]

# Supplementary information

# The Mlpt/Ubr3/Svb module comprises an ancient developmental switch for embryonic patterning

# Suparna Ray^*^, Miriam I Rosenberg^*^, Hélène Chanut-Delalande, Amelie Decaras, Barbara Schwertner, William Toubiana, Tzach Auman, Irene Schnellhammer, Matthias Teuscher, Philippe Valenti, Abderrahman Khila, Martin Klingler, and François Payre

Additional information

Supplementary file 1A-D

| **Supplementary file 1A. Genes identified in the genome wide iBeetle RNAi screen that had phenotypes resembling those of *mlpt***. | | |
| --- | --- | --- |
| **iBeetle ID #** | ***Gene model*** | **Protein function (Fly Base/BLAST)** |
| iB_04836 | *TC010440* | Ubiquilin (ubiquitin associated/proteasome-binding) |
| iB_05064 | *TC011626* | Proteasome activator pa28/ endopeptidase activity regulator |
| iB_00966 | *TC005949* | Zinc finger RING type; N-recognin (E3 ubiquitin ligase) |
| iB_09278 | *TC005948* | Zinc finger RING type; N-recognin (E3 ubiquitin ligase) |
| iB_01757 | *TC010894* | ERp60 (protein disulfide isomerase activity) |
| iB_02207 | *TC033042* | chinmo (metal-ion-/DNA-binding transcription factor) |
| iB_04057 | *TC033957* | Eph receptor tyrosine kinase |
| iB_04008 | *TC005755* | Zinc finger/RE-1 silencing transcription factor B-like |
| iB_03496 | *TC003091* | No matches. Protein has an SMC domain |
| iB_08265 | *TC010458* | Integral or transmembrane protein of unknown function |
| iB_010591 | *TC014364* | Got1 (Glutamate oxaloacetate transaminase 1) |

| **Supplementary file 1B. Oligonucleotides used in the manuscript.** | |
| --- | --- |
| **Oligo Name** | **Sequence** |
| ***Oncopeltus*** | |
| Ofas Mlpt FOR1 | GTGGGAGGCCTGTAGCGCAGGAGAGGGTACCG |
| OFAS MLPT ATG1 FOR | ATGTCTGGAGACACTCTTGACCCCACTGGTCT |
| Ofas Mlpt FL Rev 1 | GACTTTTGAATATTTAACTTCATGAAGTCTCT |
| Ofas Mlpt Stop 2 REV | TCAGTAAAGGCCTGTGGGGTCCAGAGTATCGT |
| Ofas Svb put 5UTR FOR | TTTCAAACTTTAGATAACGTAATCGGATAAAAC |
| Ofas Svb Start codon FOR | ATGCCCAAGATATTCCTGATCAAGAACCGCCTG |
| Ofas Svb 700bp shared REV | CTTCGGGACGTGGTAGTCTATAGGAGCGTCTTG |
| Ofas Ubr3 probe F | AGAGCTCAGAGTCCACAGAAGAA |
| Ofas Ubr3 probe R | TCGCAAAGATCTTAGATAAGACC |
| Ofas Ubr3 long cDNA probe F | GGTGTACATGTACAAACTTGTGGTCACCACCTTC |
| Ofas Ubr3 long cDNA probe R | CTAGAGTCCGTCTCTGTGCCAGACCCACTTCTTA |
| T7 Ofas Mlpt RNAi FOR | TAATACGACTCACTATAGGGAGACCAC ATGTCTGGAGACACTCTTGACCCCA |
| T7 Ofas Mlpt RNAi REV | TAATACGACTCACTATAGGGAGACCAC GACTTTTGAATATTTAACTTCATGA |
| T7 Ofas Svb RNAi FOR | TAATACGACTCACTATAGGGAGACCAC TTTCAAACTTTAGATAACGTAATCG |
| T7 Ofas Svb RNAi REV | TAATACGACTCACTATAGGGAGACCAC CTTCGGGACGTGGTAGTCTATAGGA |
| T7 Ofas ubr3 RNAi FOR | TAATACGACTCACTATAGGGAGACCAC AGAGCTCAGAGTCCACAGAAGAA |
| T7 Ofas ubr3 RNAi FOR | TAATACGACTCACTATAGGGAGACCAC TCGCAAAGATCTTAGATAAGACC |
| Ofas Mlpt For 1 | GTGGGAGGCCTGTAGCGCAGGAGAGGGTACCG |
| Ofas Mlpt ATG1 FOR | ATGTCTGGAGACACTCTTGACCCCACTGGTCT |
| Ofast mlpt Rev 1 | GACTTTTGAATATTTAACTTCATGAAGTCTCT |
| Ofas Mlpt stop2 REV | TCAGTAAAGGCCTGTGGGGTCCAGAGTATCGT |
| ***Nasonia*** | |
| Nv Mlpt probe FOR | ATGGCAGTGCAGCTCGACCCGACCGGTGTTTA |
| Nv Mlpt probe REV | TTTATCGAGCAGCCGGCGCTTCGTCCTCGTCT |
| Nv Svb probe FOR (FP) | ACCAGAGCCCGAACGAGCCCCAGCTTCAGCAGCT |
| Nv Svb probe REV (FP) | CGTGCGCACCTGCAGCAGCATGTTGGCGAAGTTG |
| T7 Nv UBR3 RNAi FOR | TAATACGACTCACTATAGGGAGACCAC TGTGTCATCTGCAATCAAACTAC |
| T7 Nv UBR3 RNAi REV | TAATACGACTCACTATAGGGAGACCAC TTGTCGACACAGAGGACAATTGT |
| T7 Nv Svb RNAi FOR FP | TAATACGACTCACTATAGGGAGACCAC ACCAGAGCCCGAACGAGCCCCAG |
| T7 Nv Svb RNAi REV FP | TAATACGACTCACTATAGGGAGACCAC ACGTGCGCACCTGCAGCAGCATG |
| T7 Nv mlpt RNAi FOR | TAATACGACTCACTATAGGGAGACCAC  ATGGCAGTGCAGCTCGACCCGAC |
| T7 Nv mlpt RNAi REV | TAATACGACTCACTATAGGGAGACCAC  TTTATCGAGCAGCCGGCGCTTCG |
| ***Gerris*** | |
| GB ovo/svb (dsRNA) FOR | CAGTATGGTCAAAATATGGATCAG |
| GB ovo/svb (dsRNA) REV | CTTACTTTAAACGAGGGTATACTG |
| GB ovo (dsRNA) FOR | GGTGGCCAACAAAGTTCCCT |
| GB ovo (dsRNA) REV | CCTGTGTTGAAGAACGCCAA |
| GB svb (dsRNA) FOR | ATGCCGAAGATATTTCTGATTAAA |
| GB svb (dsRNA) REV | GATGAGAGATAAAGGCTCCGGTAT |
| GB ovo/svb (probe) FOR | CCAAGCAATACTCGTTGCTACA |
| GB ovo/svb (probe) REV | GTTGTGTGTCCGCAATCCTC |
| GB pri (probe+dsRNA) FOR | TTCGTTTCTTGTTCCTACACTA |
| GB pri (probe+dsRNA) REV | AGTTTACTTTAGGGCAGTGTAA |
| GB Ubr3 (probe+dsRNA) FOR | GGTTGTATAAGATCTTCACTACTC |
| GB Ubr3 (probe+dsRNA) REV | CATGGATGATGAGTGTAAATGT |
| ***Tribolium*** | |
| ***Tc-svb* (TC008099)** | |
| E4_1_1-F | TGACACTATAGAAGTGGTAGCCGAGGTCACGTTCTC |
| E4_1_1-R | CTCACTATAGGGAGACTATCGGCTGTTCCTGTTCC |
| E4_2_1-F | TGACACTATAGAAGTGATCAATTATTGCCAGGGTCG |
| E4_2_1-R | CTCACTATAGGGAGACGAGGTTTTGGTCGACTTGT |
| E5_1_F | TGACACTATAGAAGTGGATCAAGAATCCTTTGGCGA |
| E5_1_R | CTCACTATAGGGAGACCTCGTGTGCCTCTTCAGAT |
| E6_1_F | TGACACTATAGAAGTGAAGATGTTCGTTGGAATCGC |
| E6_1_R | CTCACTATAGGGAGATCGTGAATTTGAAGTGTCGC |
| iB_06248_F | TGACACTATAGAAGTGAACTCTCCAAAACGCCTCCT |
| iB_06248_R | CTCACTATAGGGAGAGTTGTTGTTGGTGCATTTGG |
| svb2-F | AACACGCCCTTTTGGAGAGC |
| svb2-R | CTTTGGTCCGTCGTTCTTTGTAAG |
| svb_N-ter_F | TGACACTATAGAAGTGAATACGCCCCTATCGTACCC |
| svb_N-ter_R | CTCACTATAGGGAGACGAACCACCGGAAAAGTTTA |
| svb_C-term_F_SP6 | TGACACTATAGAAGTGTCGACAATTTCAACGACCAA |
| svb_C-term_R_T7 | CTCACTATAGGGAGATCGTGATCGACGCTACTTTG |
| svb1-F | AATACGCCCCTATCGTACCC |
| svb1-R | ACCCGATTGAGAATTTGTCG |
| ***Tc-ubr3*** | |
| **TC005949** | |
| E3_2_1-F | TGACACTATAGAAGTGCGAGACCGAATTCGTTTGTT |
| E3_2_1-R | CTCACTATAGGGAGACGTACTCCCTCGGCAATTTA |
| E4_1-F | TGACACTATAGAAGTGCGTGACCCACCTAATCCACT |
| E4_1-R | CTCACTATAGGGAGAGTAGCGGCAAAAGCAGGATA |
| E7_1-F | TGACACTATAGAAGTGTCGGACGAGGAGTTGTTTGT |
| E7_1-R | CTCACTATAGGGAGACAAACTCGGCACTCAACGTA |
| E8_1_1-F | TGACACTATAGAAGTGGACCATTAGCGAGCGAAAAG |
| E8_1_1-R | CTCACTATAGGGAGAATCCGGATTAACATCGGACA |
| iB_00966-F | TGACACTATAGAAGTGGCCGATTTGTATCTCCTCCA |
| iB_00966-R | CTCACTATAGGGAGACATCGACGTGTGGAAATCAC |
| **TC005948** | |
| E3_1-F | TGACACTATAGAAGTGGTGTCATGACGGATCATTGC |
| E3_1-R | CTCACTATAGGGAGAGCCCACATTGGATAAGCACT |
| E4_1_1-F | TGACACTATAGAAGTGGGACGCGGATGGTTTTATTA |
| E4_1_1-R | CTCACTATAGGGAGATCTGGAGCGAAGGACAGTCT |
| iB_09278-F | TGACACTATAGAAGTGAATGATCCCGAAAACGTCAG |
| iB_09278-R | CTCACTATAGGGAGAATGGCCAATAGCAATGATCC |
| ***Drosophila*** | |
| Svb_Fwd | GCTCGGATCCACTAGTATGGCAGCCGGACACGGACG |
| Svb_Rev | TCGATGTTGCGAATTCGGTGATGGCCAC |

| **Supplementary file 1C.** **Oligos used for generating *Tc-svb* CRISPR mutant.** | | |
| --- | --- | --- |
| Guide RNAs | Genomic target sequence | Oligos |
| gRNA1 | GGGAATAATTATAACGCAAGTGG | Sense: TTCGGGAATAATTATAACGCAAG |
|  |  | Antisense: AAACCTTGCGTTATAATTATTCC |
| gRNA2 | GCCAATTTGAGTCTAAACGGGGG | Sense: TTCGCCAATTTGAGTCTAAACGG |
|  |  | Antisense:  AAACCCGTTTAGACTCAAATTGG |
| gRNA3 | GTCTTTCATTACGATGCCGTAGG | Sense:  TTCGTCTTTCATTACGATGCCGT |
|  |  | Antisense: AAACACGGCATCGTAATGAAAGA |

| **Supplementary file 1D. Plasmids used for generating *Tc-svb* CRISPR mutant.** | |
| --- | --- |
| Plasmid | Description/Use |
| pBac(3xP3-EGFPafm) | *sim* target sequence was ligated into this vector^a^ |
| pBME(TcU6b-BsaI) | Original gRNA expression vector with *Bsa*1 sites into which *svb* gRNAs 1-3 were cloned^b^ |
| pSLfa(Hsp-p-nls-Cas9-3’UTR)fa | Cas9 expression vector^b^ |
| Tc-U6b-sim ZS1 | *sim* gRNA expression vector |

^a^ obtained from Dr. Ernst Wimmer

^b^ obtained from Dr. Anna Giles
